# Associations between estradiol and testosterone and depressive symptom scores of the Patient Health Questionnaire-9 in ovariectomized women: a population-based analysis of NHANES data

**DOI:** 10.1186/s12991-020-00315-1

**Published:** 2020-11-17

**Authors:** Ching-Yen Chen, Jian-Hong Chen, Shao-Chun Ree, Chun-Wei Chang, Sheng-Hsiang Yu

**Affiliations:** 1 Department of Psychiatry, Chang Gung Hospital, Keelung, Taiwan; 2 Faculty of Medicine, Chang Gung University, Taoyuan, Taiwan; 3 Department of Psychiatry, Fu Jen Catholic University Hospital, New Taipei, Taiwan; 4 School of Medicine, Fu Jen Catholic University, New Taipei, Taiwan; 5 Department of Psychology, Fo Guang University, No. 160, Linwei Rd., Jiaoxi, Yilan 26247 Taiwan

**Keywords:** Depression, Estradiol, Ovariectomy, Patient Health Questionnaire-9 (PHQ-9), Testosterone

## Abstract

**Background:**

Women are well known to be susceptible to developing affective disorders, yet little attention has been given to effects of ovariectomy-reduced hormones and links with depression. This population-based cross-sectional study aimed to investigate possible associations between ovariectomy-reduced hormones and depression symptom scores of the Patient Health Questionnaire-9 (PHQ-9) in ovariectomized women.

**Methods:**

Data of PHQ-9 scores, demographics and comorbidities of ovariectomized women were extracted from the U.S. National Health and Nutrition Examination Survey (NHANES) database (2013–2016) and were analyzed retrospectively.

**Results:**

Among ovariectomized women in the NHANES database, serum estradiol levels were significantly positively associated with PHQ-9 scores (*ß* = 0.014, 95% CI: 0.001, 0.028, *P *= 0.040), whereas serum testosterone was negatively associated with PHQ-9 scores (*ß* = -0.033, 95% CI: − 0.048, − 0.018, *P* < 0.001) after adjusting for confounders. Further stratified analyses revealed that serum estradiol was positively associated with PHQ-9 only among women with history of estrogen use. Serum testosterone levels were negatively associated with PHQ-9 among women with or without prior estrogen use but this was only observed among women aged <  = 60 years (*ß* = − 0.057, − 0.076, − 0.038, *P* < 0.001).

**Conclusions:**

Serum estradiol and testosterone are associated with PHQ-9 scores indicative for depression in ovariectomized women. The associations are modified by age and history of estrogen use. Future prospective studies are warranted to confirm these findings, carefully addressing possible confounding of age-related dementia.

## Background

Bilateral resection of the ovaries is sometimes performed at the time of hysterectomy for benign conditions to prevent development of ovarian cancer, accounting for 300,000 oophorectomies (ovariectomies) affecting 55% of all women in the United States [1]. The effects of ovariectomy on women’s long-term health may increase the risk of coronary artery disease and stroke, fragility fractures, Parkinsonism, cognitive impairment and dementia, and anxiety and depression [2]. Women who have undergone bilateral ovariectomy before menopause are shown to be at increased long-term risk of anxiety and depressive symptoms associated with the loss of the estrogen-producing ovaries [3]. Changes in estrogen and progesterone levels in women during specific reproductive stages are associated with an increased risk of developing depressive disorders, including premenstrual dysphoric disorder, postnatal depression, postpartum depression, and perimenopausal depression [4, 5]. A strong association also has been reported between mood fluctuations and sex hormones [6, 7]. Furthermore, it is well‐established that estrogens affect cognition, particularly learning and memory [8]. Previous studies have suggested that the most potent endogenous estrogen, 17*β*‐estradiol (known as E2), has neuroprotective properties. Low estradiol levels limit its neuroprotective ability, which may increase vulnerability to psychiatric disturbances in women [7, 9].

A previous study of elderly men and women aged 70–79 years indicated that free testosterone levels in women were associated with higher depression scores and that men with the lowest total testosterone levels had higher depression scores [10]. However, measuring the possible effects of testosterone is complicated because its metabolites have distinct functional effects. The source of estradiol-E2 in males and females is testosterone that has aromatized (although the amount of E2 made by the ovaries in females varies and may reach greater asymptotic levels than that observed in males); however, levels decline in endogenous E2 levels, or E2 ablation and replacement, in both young and aged rodents, which increases affective behaviors such as anxiety, fear, and depression [11]. Whether testosterone acts as a prohormone to increase age-related depression in both males and females remained uncertain. Further studies are required to investigate the role of testosterone and its metabolites in depression.

In ovariectomized (OVX) rats, low circulating levels of estradiol, progesterone and testosterone are used to confirm successful ovariectomy since the ovaries are the primary source of sex hormones. In addition, low uterus weight and increased body weight gain are also characteristically reported after ovariectomy [12, 13]. The levels of estradiol and testosterone in OVX rats reached only nondetectable levels [14]. Several studies have revealed that estrogen replacement therapy [15–18] was able to reverse increased anxiety levels [19, 20] and depressive-like behavior [15, 16, 19, 21] in OVX rodents.

It is well known that women are susceptible to developing affective disorders, yet little attention has been given to effects of ovariectomy-reduced hormone in the development, clinical presentation, and features of depressive disorders, along with the response to antidepressant treatments. Therefore, we conducted a population-based, cross-sectional study with the aim of evaluating associations between ovariectomy-reduced hormones and depressive symptoms in a large cohort of ovariectomized women.

## Methods

### Data source

Data for this study were obtained from National Health and Nutrition Examination Survey (NHANES) database (U.S. Department of Health and Human Services, Centers for Disease Control and Prevention), 2013–2016 [22]. This ongoing survey is conducted annually in a nationally representative sample of the non-institutionalized U.S. population (https://www.cdc.gov/nchs/nhanes/index.htm). NHANES data consist of demographic, health-related and socioeconomic information of survey participants, including age, race, ethnicity, gender, marital status, income and education [22, 23]. As such, NHANES data provide a reliable multidimensional evaluation of participants that can serve as a population-level assessment [23].

### Ethical considerations

The protocol for the present study was reviewed by the Internal Review Board of Chang Gung Memorial Hospital which exempted the study from IRB approval and waived the requirement for informed consent because all data were deidentified and patients remained anonymous.

### Study population

This cross-sectional study extracted data of women > 20 years old from the NHANES database who had undergone bilateral oophorectomy between 2013 and 2016. Those lacking results for serum testosterone or serum estradiol were excluded.

### Study variables

#### Patient Health Questionnaire-9 (PHQ-9)

Definition and assessment of depressive symptoms are similar to those in our previous study [24]. Briefly, depressive symptoms were assessed using the Patient Health Questionnaire-9 (PHQ-9), which has been recommended as a reliable and valid tool in community samples with good diagnostic sensitivity and specificity and good internal consistency for detecting depression symptoms of varying severity [25–27]. The PHQ-9 consists of nine items evaluating the presence of depressive symptoms during the prior 2 weeks. Each item of PHQ-9 is scored on a four-point Likert scale from 0 to 3 as follows: 0 (not at all), 1 (on several days), 2 (no more than half of the days), and 3 (nearly every day). Total scores of PHQ-9 range from 0 to 27 to measure severity of depressive symptoms, representing mild (score: 5), moderate (score: 10), moderately severe (score: 15), and severe depression (score: 20).

#### Serum testosterone and estradiol

According to NHANES data documentation and laboratory analytic notes in the NHANES Laboratory Procedures Manual (https://wwwn.cdc.gov/nchs/nhanes/ContinuousNhanes/Manuals.aspx?BeginYear=2013), the concentrations of serum testosterone and estradiol were measured using an isotope dilution-liquid chromatography tandem mass spectrometry (ID-LC–MS/MS) based on the reference method of the National Institute for Standards and Technology (NIST) (details can be found at: https://wwwn.cdc.gov/Nchs/Nhanes/2013-2014/TST_H.htm).

### Demographics and comorbidities

Demographic variables were obtained from questionnaire data in the NHANES database including: age (< = 60, > 60 years old), education level, family poverty income ratio, marital status. Comorbidities included diabetes, cardiovascular disease, cancer history, chronic respiratory tract disease and arthritis.

### Statistical analysis

Continuous variables are expressed as mean ± standard error, and categorical variables as counts (weighted percentage). Univariate and multivariate linear regression analyses were performed to assess associations between testosterone, estradiol and PHQ-9 scores. All significant variables found in univariate analysis were entered into multivariate analysis. In addition, stratified analyses were performed to assess associations between estradiol and testosterone levels and PHQ-9 scores according to participants’ age and history of estrogen use. All tests were applied with discharge weights to account for the NHANES sampling method, except for correlation. The NHANES database uses weights to account for the complex survey design, survey non-response, and post-stratification adjustments to match total population counts from the Census Bureau. Weighted samples in NHANES represent the U.S. civilian no institutionalized resident population (detailed information can be found at: https://wwwn.cdc.gov/nchs/nhanes/tutorials/module3.aspx). All statistical analyses were performed using SAS Version 9.4 (SAS Institute Inc., Cary, NC, USA). A value of* p* < 0.05 was considered significant.

## Results

### Participants’ characteristics

Baseline characteristics of all ovariectomized women are shown in Table 1. A total of 548 women were included with a mean PHQ-9 depressive symptom score of 4.522. Higher proportions of the study population were observed in those with age > 60 (66.36%), not poor (81.50%), education level of high school or above (88.05%), and married/living with partner (60.17%). Mean serum estradiol level was 16.175 pg/ml, and mean serum testosterone was 16.507 ng/dL. Previous estrogen use was recorded for 55.42% of women with a mean duration of 10.697 years. Among the comorbidities, arthritis had the highest proportion (65.51%) compared to other comorbidities (Table 1).

**Table 1:** Characteristics of study population

Variables	*n* = 548
PHQ-9 score	4.522 ± 0.316
Demographics	
Age	
< = 60	166 (33.64%)
> 60	382 (66.36%)
Poverty income ratio^a^	
Not poor	412 (81.50%)
Poor	94 (11.98%)
Education level	
Below high school	110 (11.95%)
High school or above	438 (88.05%)
Married/live with partner	
No	270 (39.83%)
Yes	278 (60.17%)
Serum estradiol, pg/ml	16.175 ± 1.981
Serum testosterone, ng/dL	16.507 ± 1.328
History of estrogen use	
No	18 (4.92%)
Yes	267 (55.42%)
Duration of estrogen use (years)	10.697 ± 1.108
Comorbidities	
Diabetes	
No	411 (78.82%)
Yes	137 (21.18%)
CVD	
No	439 (81.07%)
Yes	109 (18.93%)
Cancer history	
No	409 (71.61%)
Yes	139 (28.39%)
Chronic respiratory tract disease	
No	431 (76.40%)
Yes	117 (23.60%)
Arthritis	
No	206 (34.49%)
Yes	342 (65.51%)

Continuous variables are presented as mean ± standard error (SE); categorical variables are presented as counts (weighted percentage)

Numbers may not add up to 100% due to missing value *CVD* cardiovascular disease

^a^Poverty income ratio for not poor was defined as ≥ 1, and for poor was defined as < 1

### Associations between PHQ-9 scores and study variables

Results of univariate analysis showed that education level, serum testosterone, estrogen use duration, CVD, chronic respiratory tract disease, and arthritis were significantly associated with PHQ-9 scores. After adjustments for multivariate analysis, serum estradiol (*ß* = 0.014, 95% CI: 0.001, 0.028) was significantly positively associated with PHQ-9 scores, whereas serum testosterone (*ß* = − 0.033, 95% CI: -0.048, -0.018) and duration of estrogen use (*ß* = − 0.11, 95% CI: − 0.147, − 0.072) were significantly negatively associated with PHQ-9 scores (Table 2).

**Table 2:** Associations between PHQ-9 scores and study variables

Variables	Univariate			Multivariate		
	*ß*	95% CI	*P* value	*ß*	95% CI	*P* value
Demographics						
Age						
< = 60	REF	REF	REF	–	–	–
> 60	− 1.113	(− 2.701, 0.475)	0.163	–	–	–
Poverty income ratio^a^						
Not poor	Ref	Ref	Ref	–	–	–
Poor	2.189	(− 0.321, 4.699)	0.085	–	–	–
Education level						
Below high school	Ref	Ref	Ref	Ref	Ref	Ref
High school or above	*− 2.189*	*(− 4.370, -0.009)*	*0.049*	− 2.150	(− 5.709, 1.409)	0.227
Married/live with partner						
No	Ref	Ref	Ref	–	–	–
Yes	− 0.213	(− 1.391, 0.965)	0.715	–	–	–
Serum estradiol, pg/ml	− 0.003	(− 0.011, 0.004)	0.378	*0.014*	*(0.001, 0.028)*	*0.040*
Serum testosterone, ng/dL	*− 0.021*	*(− 0.034, − 0.008)*	*0.003*	*− 0.033*	*(− 0.048, − 0.018)*	* < 0.001*
History of estrogen use						
No	Ref	Ref	Ref	–	–	–
Yes	1.096	(-0.198, 2.390)	0.094	–	–	–
Duration of estrogen use (years)	*− 0.104*	*(− 0.148, -0.060)*	* < 0.001*	*− 0.110*	*(− 0.147, − 0.072)*	* < 0.001*
Comorbidities						
Diabetes						
No	Ref	Ref	Ref	–	–	–
Yes	1.641	(− 0.041, 3.322)	0.055	–	–	–
CVD						
No	Ref	Ref	Ref	Ref	Ref	Ref
Yes	*3.093*	*(1.552, 4.634)*	* < 0.001*	0.684	(− 1.063, 2.431)	0.430
Cancer history						
No	Ref	Ref	Ref	–	–	–
Yes	0.165	(− 0.976, 1.307)	0.769	–	–	–
Chronic respiratory tract disease						
No	Ref	Ref	Ref	Ref	Ref	Ref
Yes	*2.754*	*(0.934, 4.575)*	*0.004*	1.254	(− 0.604, 3.113)	0.178
Arthritis						
No	Ref	Ref	Ref	Ref	Ref	Ref
Yes	*2.024*	*(0.994, 3.053)*	* < 0.001*	0.949	(− 0.335, 2.233)	0.141

Significant values are shown in italic (*p* < 0.05)

### Associations between estradiol and testosterone and PHQ-9 scores by age and history of estrogen use

After stratifying by age, the results revealed that estradiol was positively associated with PHQ-9 scores only among women with a history of estrogen use (*ß* = 0.014, 95% CI: 0.001, 0.027). However, serum estradiol was not significantly associated with PHQ-9 scores when stratified by age groups. Serum testosterone was significantly negatively associated with PHQ-9 scores (*ß* = − 0.057, 95% CI: − 0.076, − 0.038) among women aged ≤ 60 years. Testosterone was also significantly negatively associated with PHQ-9 scores, regardless of whether it was with (*ß* =− 0.028, 95% CI: − 0.042, − 0.014) or without (*ß* = − 0.186, 95% CI: − 0.331, − 0.040) history of estrogen use (Table 3).

**Table 3:** Associations between PHQ-9 scores, serum estradiol and testosterone by age and history of estrogen use

Term		PHQ-9 score		
		*ß*	95% CI	*P* value
Serum estradiol, pg/ml				
Age^a^	< = 60 (*n *= 166)	0.015	(− 0.054, 0.084)	0.663
	> 60 (*n* = 382)	0.007	(− 0.009, 0.022)	0.383
History of estrogen use^b^	No (*n* = 18)	0.013	(− 0.004, 0.031)	0.136
	Yes (*n* = 267)	*0.014*	*(0.001, 0.027)*	*0.036*
Serum testosterone, ng/dL				
Age^a^	< = 60 (*n* = 166)	*− 0.057*	*(− 0.076, − 0.038)*	* < 0.001*
	> 60 (*n* = 382)	− 0.004	(− 0.052, 0.045)	0.876
History of estrogen use^b^	No (*n* = 18)	*− 0.186*	*(− 0.331, − 0.040)*	*0.014*
	Yes (*n* = 267)	*− 0.028*	*(− 0.042, − 0.014)*	* < 0.001*

Significant values are shown in italic (*p* < 0.05)

^a^Adjusted for education level, serum estradiol (pg/ml), serum testosterone (ng/dL), estrogen use duration (years), *CVD* chronic respiratory tract disease, arthritis

^b^Adjusted for education level, serum estradiol (pg/ml), serum testosterone (ng/dL), *CVD* chronic respiratory tract disease, arthritis

## Discussion

In this cross-sectional, population-based study, evaluation of associations between serum estradiol and testosterone and depression symptom scores of the PHQ in ovariectomized women revealed that serum estradiol was positively associated with PHQ-9 scores, whereas serum testosterone levels were negatively associated with PHQ-9 scores. However, these associations were moderated by age and history of estrogen use.

Previous studies have revealed that women who have their ovaries removed (ovariectomy) have an increased risk of depression, which imitates spontaneous or surgically induced menopause [28, 29]. Ovariectomy in rodents is a well-established surgical menopause model for mimicking the loss of ovarian hormones in humans [30, 31], resulting in an increased risk of depressive behaviors [14, 32].

Estrogen replacement therapy for the treatment of menopausal somatic symptoms has been long-reported to improve subjective well-being and quality of life during perimenopause [33–35]. An earlier study by Schmidt et al. [36] found that the use of 17*β*-estradiol (50 µg) is beneficial for mood improvement in women who meet the criteria for perimenopause-onset major or minor depression. Soares et al. [37] reported that 67% of women treated with estradiol had remission of depression and continued mood improvement in a 12-week treatment period. In our analyses, a history of estrogen use was not significantly associated with depression-related symptom scores. However, when considering women who had history of estrogen use, the results demonstrated that longer duration of estrogen use was significantly associated with lower PHQ-9 scores which indicates the presence of fewer depression-related symptoms, or lower severity of depressive symptoms.

The hippocampus is central in the pathophysiology and etiology of depression hippocampal volumes were found to be reduced in depressed patients [38], and this reduction is shown to be prevented and reversed by treating with antidepressants [39]. Patients with increased levels of glucocorticoids have also been shown to exhibit the depression-like symptomatology, and hypothalamus–pituitary–adrenals (HPA) axis dysfunction was found in depressed patients, indicating a link between glucocorticoids and affective states [40]. Earlier study also established that gonadal hormones influence glucocorticoid levels [41] and that depression correlates strongly with glucocorticoids when patients have comorbid anxiety [42]. A recent study reported that neurogenesis in the hippocampus is improved by the activation of the estrogen receptor (ER) [43]. Study in a rat model also demonstrated that 2–4 days after withdrawal of estrogen benzoate (EB)-stimulated hormone-simulated pregnancy (HSP) in ovariectomized rats, depression-like behaviors were presented in these HSP rats [44, 45].

In the present study, the descriptive statistics shows 66.36% of ovariectomized women were over 60 years old. Given this relatively high proportion of elderly women, indices of depressive-related symptoms which might overlap with dementia symptoms may confound the results. However, the NHANES data lacked definitive diagnosis of dementia or cognitive assessment for participants, which limited our analysis. We have tried to reduce this possible confounding effect by performing stratified analyses according to age (and use of estrogen or not), analyzing younger and elder ages separately. The results of stratified analysis showed serum testosterone and PHQ-9 scores was associated regardless of whether it was with or without a history of estrogen use, but the association was only observed among ovariectomized women aged ≤ 60 years. The underlying mechanism of these associations cannot be evaluated by the current study design. However, based on the aforementioned literature, we suggest that the associations between serum levels of testosterone and estradiol and depressive symptom severity may be due to regulation of ER/hippocampus signaling in ovariectomized women. Further investigations need to be conducted to determine whether the molecular mechanism of testosterone- and estradiol-related depression is influenced by the regulation of ER/hippocampus signaling.

## Limitations

The present study has several limitations, including its retrospective, cross-sectional design, which does not allow inferences of causality or determination of depression etiology. Although the NHANES database is a representative sample of the United States population and results can be generalized across the national population, reporting bias still may occur in the self-assessed questionnaire data used. Also, the NHANES database lacked definitive diagnosis of dementia, which limited our evaluation and discussion of possible confounding of results by age, particularly when considering age-related dementia that could influence findings relative to depression in older participants. Findings of the present study should be validated by longitudinal studies in different populations.

## Conclusion

Serum estradiol and serum testosterone are associated with PHQ-9 scores in ovariectomized women. These associations are modified by age and history of estrogen use. Future prospective studies are warranted to confirm these findings, with confounding from age-related dementia carefully addressed.

## Data Availability

All data generated within this study are available from the corresponding author on request.

## Abbreviations

CVD: Cardiovascular disease
EB: Estrogen benzoate
ER: Estrogen receptor
HSP: Hormone-simulated pregnancy
HPA: Hypothalamus–pituitary–adrenals
MDD: Major depressive disorder
NHANES: National Health and Nutrition Examination Survey
OVX: Ovariectomized
PHQ-9: Patient Health Questionnaire-9
